# Rapidly evolving conjunctivitis due to *Pasteurella multocida*, occurring after direct inoculation with animal droplets in an immuno-compromised host

**DOI:** 10.1186/s12886-015-0002-6

**Published:** 2015-03-08

**Authors:** Anthony Corchia, Anne Limelette, Béatrice Hubault, Ailsa Robbins, Anne Quinquenel, Firouze Bani-Sadr, Yohan N’Guyen

**Affiliations:** Service de Médecine Interne, Maladies Infectieuses et Immunologie Clinique, Hôpital Robert Debré, Avenue Général Koenig, 51100 Reims, France; Laboratoire de Bactériologie, Hôpital Robert Debré, Avenue Général Koenig, 51100 Reims, France; Service d’Ophtalmologie, Hôpital Robert Debré, Avenue Général Koenig, 51100 Reims, France; Service d’Hématologie Clinique, Hôpital Robert Debré, Avenue Général Koenig, 51100 Reims, France

**Keywords:** *Pasteurella*, Conjunctivitis, Immuno-compromised, Droplet, Oxidase test

## Abstract

**Background:**

The rare descriptions, in the literature, of ocular infections due to *Pasteurella multocida* include: endophtalmitis, keratitis and corneal ulcers, Parinaud’s oculoglandular syndrome, and conjunctivitis. Here, we report a rare case of rapidly evolving conjunctivitis due to *Pasteurella multocida*, occurring after direct inoculation with animal droplets in an immuno-compromised host.

**Case presentation:**

A 69-year-old, Caucasian male was referred to our department with purulent conjunctivitis, occurring five days after chemotherapy for an angioimmunoblastic-T-cell-lymphoma, and thirty-three hours after being struck in his right eye by his sneezing Dachshund dog. Physical examination revealed purulent conjunctivitis of the right eye associated with inflammatory edema of both lids. Direct bacteriological examination of conjunctival secretions showed gram-negative bacilli and regular, grey non-hemolytic colonies appearing the next day on blood agar. The oxidase test was positive for these colonies. An antibiotherapy associating intravenous amoxicillin and amoxicillin/clavulanate was administered. The outcome was favorable in the next three days allowing discharge of the patient with amoxicillin (2 g tid per os).

**Conclusion:**

This case report may be of interest for infectious diseases, ophthalmology or oncology specialists, especially nowadays with chemotherapy being administered in day care centres, where unusual home pathogens can be encountered in health related infections. In this case, previous animal contact and conjunctival samples showing *Enterobacteriaceae* like colonies with positive oxidase test were two important clues which could help clinicians to make the diagnosis of *Pasteurella* conjunctivitis in every day practice.

## Background

*Pasteurella multocida, “*killer of multiple species” [1], is a gram-negative rod that is common in the oropharyngeal microflora of numerous animal hosts. It is responsible for fowl cholera in birds and hemorrhagic fever in cattle [2]. In humans, *Pasteurella multocida* is a common causative agent of dermohypodermitis, tenosynovitis and septic arthritis in immuno-competent and immuno-compromised hosts, usually after dog/cat bites or scratches [2,3]. Device related infections or post-surgical infections due to *Pasteurella multocida* have also been reported [1,4], but ocular infections due to this bacterium have rarely been described. Indeed, human ocular infections due to *Pasteurella* genus, including *Pasteurella multocida,* that are unrelated to animal bites/scratches, have been described in only 12 out of the 3699 infections in a 12-year long British study [5], and 4 out of the 136 human infections in a 3-year long American study [6]. Ocular infections due to *Pasteurella multocida,* reported in the literature, include endophtalmitis [2,7,8], keratitis and corneal ulcers [6], Parinaud’s oculoglandular syndrome [9] and conjunctivitis [10-12].

Here, we report a rare case of rapidly evolving conjunctivitis due to *Pasteurella multocida*, occurring after direct inoculation with animal-derived droplets in an immuno-compromised host.

## Case presentation

**Figure 1:**
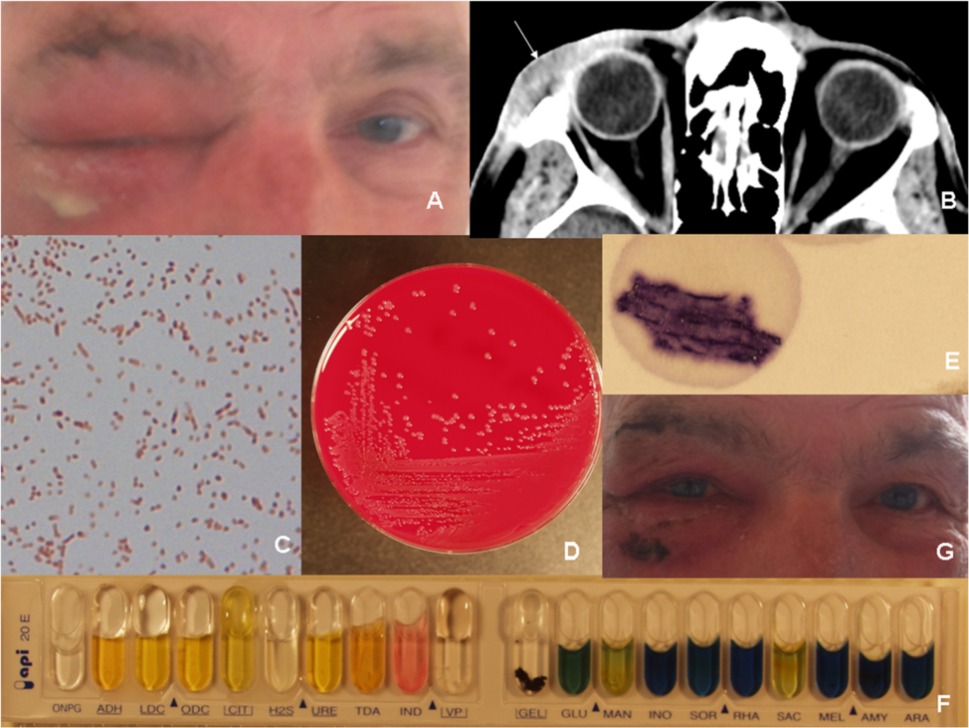
**Clinical evolution and results of bacteriological analysis of our patient. A**: Inflammatory oedema of both upper and lower lids with purulent encystment below the right eye, 33 hours after inoculation. The patient is unable to open his right eye. **B**: Computerized tomography scan with intravenous injection of contrast medium evidencing the absence of intra-orbital extension. White arrow shows pre septal inflammatory oedema. **C**: Gram staining of conjunctival samples evidencing gram-negative bacilli. **D**: Regular, smooth, grey, non hemolytic colonies cultured on blood agar. **E**: Positive oxydase test with dark blue coloration of the reagent inoculated with previously observed colonies. A negative result (e.g *Enterobacteriaceae*) would have been associated with the absence of coloration of the reagent. **F**: API-20E array results. **G**: Regression of inflammatory oedema of both upper and lower lids after 3 days of antibiotherapy associating amoxicillin 2 g tid and amoxicillin/clavulanate 2 g tid.

A 69-year-old Caucasian male was referred to our department for purulent conjunctivitis. He had no past medical history except for an angioimmunoblastic-T-cell-lymphoma, currently treated with chemotherapy, including lenalidomide and CHOP (Cyclophosphamide, Hydroxydaunorubicin, Vincristine, and Prednisone). Five days after his third round of chemotherapy, which he received in day hospital, his Dachshund dog struck his right eye while sneezing. The patient did not feel any pain and confirmed that his dog had not bitten him. Four hours later, his wife noticed redness around his eye and an inflammatory peri-orbital edema, associated with fever reaching 39°C, appeared during the following night. He took no medication. The patient was unable to open his right eye. Upon admission, 33 hours later, physical examination, revealed purulent conjunctivitis with chemosis and conjunctival hyperemia of the right eye associated with inflammatory edema of both the upper and lower lids. Skin rupture and purulent encystment were observed above and below his eye respectively (Figure 1A). The visual acuity was 3/10 in the right eye and 4/10 in the left eye. Slit lamp instrument examination showed bilateral corticonuclear cataract. Intraocular pressure was 10 mm Hg and the fluorescein eye test were normal in the left eye. The latter tests could not be performed on the right eye. There were neither relative afferent pupillary defects nor extraocular movement abnormalities. The rest of the physical examination was unremarkable. The white blood-cell count was normal (4900/mm3) but the differential revealed lymphopenia (300/mm3). The absolute neutrophil count was within normal range (4400/mm3). A computerized tomography scan revealed no evidence of intra-orbital extension (Figure 1B). Blood cultures were negative. Conjunctival secretions were sampled on admission for bacteriological analysis and cultured on blood agar. Direct examination showed numerous leukocytes and rare gram-negative bacilli (Figure 1C). Regular, grey, smooth colonies grew the next day. The oxidase test was positive for the 1-3 mm diameter, non-hemolytic colonies (Figure 1D&E), strongly suggesting that the isolated strain belonged to *Pasteurella genus*. The involvement of *Pasteurella multocida* was also strongly suggested here by mannitol fermentation (API-20E Array) (Figure 1F) and confirmed by MALDI TOF mass spectrometry. The diagnosis of *Pasteurella multocida* conjunctivitis with pre septal-cellulitis was therefore retained. Taking into account the animal contact and the short incubation period, suggesting the involvement of *Pasteurella* genus, an antibiotherapy associating amoxicillin 2 g tid and amoxicillin/clavulanate 2 g tid was administered intravenously, immediately after bacteriological sampling. No topical therapy was prescribed excepted for artificial tears. The outcome was favourable in the next three days (Figure 1G), allowing discharge of the patient with amoxicillin (2 g tid per os) alone once usual full sensitivity of *Pasteurella multocida* strain to amoxicillin was confirmed.

Herein, we report a rare case of rapidly evolving conjunctivitis due to *Pasteurella multocida*, unrelated to animal bite, occurring after direct inoculation with animal-derived droplets, in a host who was immuno-compromised after chemotherapy in daycare centre. The key element in this diagnosis was the animal contact spontaneously described by the patient rather than the classical short incubation time between animal contact and first symptoms [2]. This short incubation time has not clearly been reported in previous conjunctivitis case reports [10-12]. In this case we cannot conclude whether this rapidly evolving conjunctivitis was due to the immuno-compromised state of our patient or to the pathogenic potential of the *Pasteurella multocida* strain itself.

To rapidly assess the presence of such a pathogen, conjunctival secretions were sampled for bacteriological analysis, as suggested by a recent Turkish study that evidenced *Pasteurella canis* in 4 out of 13 cases of bacterial conjunctivitis resistant to empirical topical antibiotherapy with no history of animal contact [13]. The presence of smooth *Enterobacteriaceae-*like colonies with positive oxidase test argued in favor of *Pasteurella* genus. Moreover the involvement of *Pasteurella multocida* was confirmed by the presence of mannitol fermentation using the API20E Gallery [14] and MALDI TOF mass spectrometry. This bacteriological diagnosis allowed us to validate an antibiotherapy regimen consisting mainly of amoxicillin, which is the drug of choice against *Pasteurella* strains even if it is unusual in post chemotherapy fever [15]. Indeed, *Pasteurella* strains are usually fully sensitive to narrow spectrum antibiotics like ampicillin and amoxicillin [14] and reports of penicillinase-producing strains are rare [4]. Here, antibiotherapy associating amoxicillin 2 g tid and amoxicillin/clavulanate 2 g tid was immediately administered intravenously, once bacteriological sampling had been performed, in order to cover all *Pasteurella* species (including penicillinase-producing strains) as well as staphylococci, streptococci and anaerobes that can be encountered after dog bites [16]. Moreover, such an antibiotherapy was prescribed, despite its poor ocular diffusion, because conjunctivitis without eye involvement was here considered as an infection of soft tissues (such as cellulitis), for which amoxicillin and amoxicillin/clavulanate are strongly recommended [16].

## Conclusion

From our point of view, this case report may be of interest for infectious diseases, ophthalmology or oncology specialists nowadays (i) at a time of nationwide antibiotic sparing campaigns and (ii) at a time of ambulatory chemotherapy or chemotherapy in day care centres. The case reported here is an illustration that unusual home pathogens can be encountered in health related infections during these novel chemotherapy processes. In this case, animal contact and conjunctival samples giving *Enterobacteriaceae-*like colonies with positive oxidase test were two important clues which could help clinicians to make the diagnosis of *Pasteurella* conjunctivitis in every day practice.

## Consent

Written informed consent was obtained from the patient for publication of this Case report and any accompanying images. A copy of the written consent is available for review by the Editor of this journal.

## References

[1] Guion TL, Sculco TP (1992). *Pasteurella multocida* Infection in Total Knee Arthroplasty. J Arthroplasty.

[2] Weber DJ, Wolfson JS, Swartz MN, Hooper DC (1984). *Pasteurella multocida* infections: report of 34 cases and review of the literature. Medicine (Balt).

[3] Arons MS, Fernando L, Polayes IM (1982). Pasteurella multocida–the major cause of hand infections following domestic animal bites. J Hand Surg [Am].

[4] Sol PM, van de Kar NC, Schreuder MF (2013). Cat induced Pasteurella multocida peritonitis in peritoneal dialysis: a case report and review of the literature. Int J Hyg Environ Health.

[5] Young SEJ (1988). Pasteurella Infections 1976-1986. PHLS Microbiol Dig.

[6] Hubbert WT, Rosen MN (1970). Pasteurella multocida infection in man, unrelated to animal bite. Am J Public Health.

[7] Hoffman ME, Sorr EM, Barza M (1987). Pasteurella multocida endophtalmitis. Br J Ophtalmol.

[8] Vartian CV, Septimus EJ (1989). Endophtalmitis due to Pasteurella multocida and CDC EF-4. J Infect Dis.

[9] Balster L, Bopp S (1987). Oculoglandular syndrome (Parinaud) caused by Pasteurella multocida with corneal involvement. A severe clinical course. Fortschr Ophthalmol.

[10] Eschete M, Rambin ED, West BC (1978). *Clostridium pseudotetanicum* bacteremia in a patient with Pasteurella multocida conjunctivitis. J Clin Microbiol.

[11] Tharmaseelan K, Morgan MS (1993). *Pasteurella multocida* conjunctivitis. Br J Ophtalmol.

[12] McNamara MP, Richie M, Kirmani N (1988). Ocular infections secondary to *Pasteurella multocida*. Am J Ophthalmol.

[13] Balikoglu-Yilmaz M, Yilmaz T, Esen AB, Engin KN, Taskapili M (2012). Pasteurella canis and Granulicatella adjacens conjunctivitis outbreak resistant to empirical treatment in a child welfare agency. J Pediatr Ophthalmol Strabismus.

[14] Donnio PY (2007). Pasteurella. Freney Précis de bactériologie clinique.

[15] Freifeld AG, Bow EJ, Sepkowitz KA, Boeckh MJ, Ito JI, Mullen CA (2011). Clinical practice guideline for the use of antimicrobial agents in neutropenic patients with cancer: 2010 update by the infectious diseases society of america. Clin Infect Dis.

[16] Stevens D, Bisno AK, Chambers HF, Dellinger P, Goldstein EJC, Gorbach SL (2014). Practice Guidelines for the Diagnosis and Management of Skin and Soft Tissue Infections: 2014 Update by the Infectious Diseases Society of America. Clin Infect Dis.

